# Korean Red Ginseng Improves Blood Pressure Stability in Patients with Intradialytic Hypotension

**DOI:** 10.1155/2012/595271

**Published:** 2012-05-08

**Authors:** I-Ju Chen, Ming-Yang Chang, Sheng-Lin Chiao, Jiun-Liang Chen, Chun-Chen Yu, Sien-Hung Yang, Ju-Mei Liu, Cheng-Chieh Hung, Rong-Chi Yang, Hui-Chi Chang, Chung-Hua Hsu, Ji-Tseng Fang

**Affiliations:** ^1^Center for Traditional Chinese Medicine, Chang Gung Memorial Hospital, Taoyuan 333, Taiwan; ^2^Branch of Chinese Medicine and Linsen, Taipei City Hospital, Taipei 103, Taiwan; ^3^Department of Nephrology, Kidney Research Center, Chang Gung Memorial Hospital, Chang Gung University College of Medicine, 5 Fu-Shin Street, Kueishan, Taoyuan 333, Taiwan; ^4^Chang Gung University College of Medicine, Taoyuan 333, Taiwan; ^5^Department of Internal Medicine, Taipei Medical University Hospital, Taipei 110, Taiwan; ^6^Departments of Nuclear Medicine, Chang Gung Memorial Hospital, Taoyuan 333, Taiwan

## Abstract

*Introduction*. Intradialytic hypotension (IDH) is a common complication during hemodialysis which may increase mortality risks. Low dose of Korean red ginseng (KRG) has been reported to increase blood pressure. Whether KRG can improve hemodynamic stability during hemodialysis has not been examined. *Methods*. The 8-week study consisted of two phases: observation phase and active treatment phase. According to prehemodialysis blood pressure (BP), 38 patients with IDH were divided into group A (BP ≥ 140*/*90 mmHg, *n* = 18) and group B (BP < 140*/*90 mmHg, *n* = 20). Patients were instructed to chew 3.5 gm KRG slices at each hemodialysis session during the 4-week treatment phase. Blood pressure changes, number of sessions disturbed by symptomatic IDH, plasma levels of vasoconstrictors, blood biochemistry, and adverse effects were recorded. *Results*. KRG significantly reduced the degree of blood pressure drop during hemodialysis (*P* < 0.05) and the frequency of symptomatic IDH (*P* < 0.05). More activation of vasoconstrictors (endothelin-1 and angiotensin II) during hemodialysis was found. The postdialytic levels of endothelin-1 and angiotensin II increased significantly (*P* < 0.01). *Conclusion*. Chewing KRG renders IDH patients better resistance to acute BP reduction during hemodialysis via activation of vasoconstrictors. Our results suggest that KRG could be an adjuvant treatment for IDH.

## 1. Introduction

Intradialytic hypotension (IDH) is a common complication which occurs in 20–30% of all dialysis treatments [1]. It has a negative impact on quality of life due to associated symptoms such as nausea, vomiting, dizziness, and cramps. IDH may cause premature discontinuation of hemodialysis that may lead to chronic underdialysis and higher mortality risks [1, 2]. 

The mechanism responsible for IDH is an inappropriate response of cardiovascular and neurohormonal systems to the acute plasma volume removal during dialysis [3–5]. Hemodialysis prescriptions include ultrafiltration amount, dialysate composition, and solution temperature may affect the frequency of IDH [6–8]. Older age, ischemic heart disease, diabetes, and autonomic neuropathy increase the risk of developing IDH [9–11]. Common interventions to prevent IDH include adaptation of dialysis prescriptions, avoidance of food during dialysis, and administration of vasoconstrictor agents (e.g., midorine, adenosine antagonist) [1, 6, 12–14].

*Panax ginseng*, a naturally occurring compound that has been used for several thousand years in the orient [15], is one of the most popular herbs in the world due to its therapeutic effects on modulating immune and cardiovascular functions [16]. Previous studies on *Panax ginseng* have confirmed its effect on regulating blood pressure [15]. Interestingly, *Panax ginseng* at low-doses can elevate blood pressure, while high-dose *Panax ginseng* has hypotensive effect in healthy subjects [17–19]. However, the potential therapeutic effect of *Panax ginseng* in patients with IDH has not been examined.

Korean red ginseng (KRG), a steamed form of *Panax ginseng* with preserved major constituents, has been shown to possess more biological activity than *Panax ginseng* [20–22]. Here we conducted a prospective study to evaluate the effects of KRG on the occurrence of symptomatic IDH during hemodialysis. We also examined the changes of endothelin-1 (ET-1), plasma renin activity (PRA), angiotensin II (AngII), and nitric oxide (NO) products during hemodialysis with and without oral KRG administration.

## 2. Material and Methods

### 2.1. Participants

**Figure 1:**
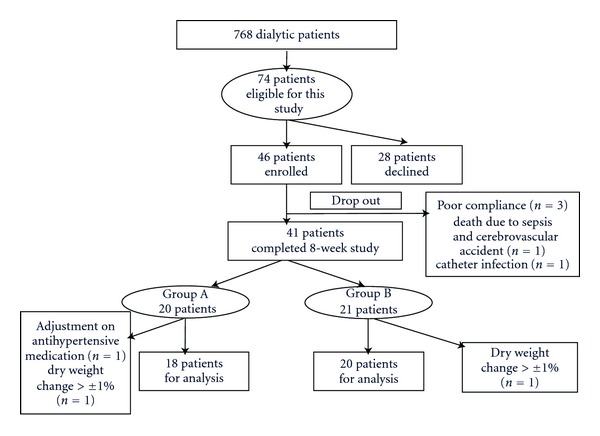
Flow chart of patient recruitment. Patients aged from 20 to 80 year old on thrice-weekly hemodialysis, with a treatment time of at least 180 minutes and had at least three symptomatic episodes of intradialytic hypotension (IDH) in the 30 days preceding enrollment, were enrolled in the study.

 The study was conducted at the hemodialysis center in Taoyuan Chang Gung Memorial Hospital (TCGMH), Taiwan. The study was approved by Chang Gung Memorial Hospital Ethical Review Committees. Each patient signed informed consent before enrollment. Patients aged from 20- to 80-year-old on thrice-weekly hemodialysis, with a treatment time of at least 180 minutes, and had at least three symptomatic episodes of IDH in the 30 days preceding enrollment, were enrolled in the study. IDH was defined as a decrease in systolic blood pressure (≥20 mmHg) or a decrease in mean arterial pressure (≥10 mmHg) accompanied by symptoms (dizziness, cramps, or fatigue, etc.), according to Kidney Disease Outcomes Quality Initiative Clinical Practice Guidelines for Cardiovascular Disease in Dialysis Patients [23]. The exclusion criteria included pregnancy or breast feeding, active infectious disease, current intake of antihypotensive medication or warfarin, and frequent changes of dry body weight (>±1% during the screening phase) and severe medical conditions included liver cirrhosis, heart failure, autoimmune disease, and cancer. Of the 768 patients receiving chronic dialysis in our hospital, 74 patients were eligible for this study; 46 patients were enrolled, while 28 patients declined to participate (Figure 1).

### 2.2. Study Design

The prospective study was designed to evaluate the pre- and posttreatment differences, and each patient served as his/her own control. The 8-week study consisted of two phases: an observation phase (phase I) and an active treatment phase (phase II). Each phase was composed of twelve consecutive HD treatments (4 weeks). Patients were given standard treatment for IDH during phase I. These enrolled patients were divided into two groups according to their prehemodialysis blood pressure. Patients with average prehemodialysis blood pressure more than 140 mmHg systolic or 90 mmHg diastolic during phase I were clustered to group A, while patients with normal or low prehemodialysis blood pressure to group B.

### 2.3. Treatment

During the active treatment phase (phase II), patients were instructed to chew 3.5 gm KRG slices at each hemodialysis session. Each slice of KRG was put into the mouth until melted, then chewed and swallowed. Patients in group A (hypertensive at baseline) were given KRG slices 60 minutes after the onset of hemodialysis to prevent late onset of hypotension after ultrafiltration. In contrast, patients in group B (normotensive or hypotensive at baseline) were given KRG slices 30 minutes before hemodialysis to prevent early onset of hypotension [24]. Cheong-Kwan-Jang Korean red ginseng (Korea Ginseng Corporation; Korea) was used in this study. The active constituents of KRG are ginsenosides including Rb1 (1.96%), Rb2 (2.18%), Rc (1.47%), Rd (0.72%), Re (1.11%), Rf (0.24%), Rg1 (0.49%), Rg2 (0.13%), Rg3 (0.12%), Rh1 (0.12%), and Rh2 (0.003%) [25]. Routine dialysis prescriptions including dialyzer types, dialysate compositions, dialysate temperature, dialysis frequencies, treatment time, and antihypertensive medications were maintained constantly throughout two study phases.

### 2.4. Outcome Parameters

Arterial blood pressure was measured with an electronic digital sphygmomanometer every 60 minutes from the beginning to the end of each dialysis session. In the event of IDH, the blood pressure was checked every 10 minutes. Prehemodialysis (pre-HD), posthemodialysis (post-HD), and intradialytic lowest (nadir) blood pressure at each dialysis session were recorded. The difference of SBP, DBP, and MAP between prehemodialysis and nadir, and between prehemodialysis and posthemodialysis, were calculated. The number of sessions disturbed by symptomatic IDH was defined as hypotensive episodes that require medical intervention including transient reduction or premature stop of ultrafiltration, infusion of isotonic or hypertonic saline, or glucose solution. These parameters during each study phase were recorded by the nurses at the hemodialysis center. Any adverse effects that might be related to KRG were recorded.

### 2.5. Assays

In the last dialysis sessions of each study phase, plasma levels of ET-1, PRA, AngII, and NO products were checked before and after dialysis. Blood samples were collected with prechilled polypropylene tubes containing 1 mg/mL of K2-EDTA and 500 KIU/mL aprotinin (Sigma). Plasma was separated by centrifugation at 3000 rpm for 15 minutes at 4°C and stored at −70°C until assayed. Specific antibodies and radioimmunoassay kits were used to assay PRA (DiaSorin, MN, USA), AngII (Phoenix Pharmaceuticals Inc. USA), and ET-1 (Peninsula Laboratories, LLC, USA). The final products of NO metabolism were examined using an assay for detecting the plasma levels of the nitrate+nitrite (NT) (Cayman Chemical Company, Ann Arbor). Hematocrit, albumin, electrolytes, and alanine aminotransferase were monitored at the beginning and end of the study by autoanalyzer. 

### 2.6. Statistical Analyses

All numerical values are expressed as mean±standard deviation (SD). Baseline variables are compared with the *χ* ^2^ test for dichotomous variables. The Mann-Whitney *U* test is applied to compare intergroup differences. The Wilcoxon signed-rank test is used for intragroup comparison between phase I and phase II. A two-tailed *P* < 0.05 is considered statistically significant. Statistical analysis is performed with analytical Software SPSS 12.0 version.

## 3. Results

### 3.1. Patient Characteristics

Of the 46 enrolled patients, 21 patients were grouped into group A and 25 patients to group B according to prehemodialysis blood pressure in the observation phase (phase I) (Figure 1). In total, 41 patients completed the KRG treatment phase (phase II), and five patients dropped out of the study prematurely due to poor compliance (*n* = 1 in group A; *n* = 2 in group B), catheter infection (*n* = 1 in group B), and death due to sepsis and cerebrovascular accident (*n* = 1 in group A) which was not directly related to KRG use. For the statistical analyses, three patients were excluded due to adjustment of antihypertensive medications (*n* = 1 in group A) and changes of dry weight exceeding ±1% (*n* = 2 in group B). Finally, 18 patients in the group A and 20 patients in the group B were included for analysis.

Baseline patient characteristics of the 38 patients of the analysis population are listed in Table 1. The mean age was  53 ± 12  years; 25 (65.8%) were females and 13 (34.2%) were males. Compared to patients in group B (normal or low prehemodialysis BP), patients in group A (high prehemodialysis BP) had a significantly shorter average time on maintenance dialysis (68 ± 49 versus  116 ± 65  months, *P* = 0.016) and a higher percentage of diabetes mellitus (72.2% versus 10%, *P* < 0.001).

### 3.2. Effect of KRG on Symptomatic IDH

**Figure 2:**
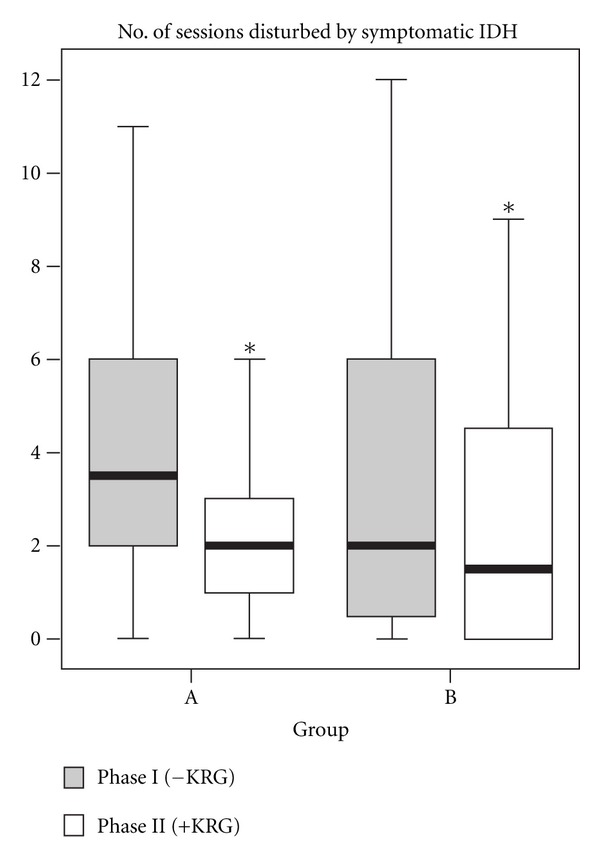
Comparison of the frequency of intradialytic hypotension (IDH) with and without Korean red ginseng (KRG) treatment. Phase I is the control phase (grey boxes). Phase II is the KRG treatment phase (white boxes). The number of sessions disturbed by symptomatic IDH reduced significantly after KRG treatment in both group A (hypertensive at baseline, *n* = 18, *P* = 0.016) and group B (normotensive or hypotensive at baseline, *n* = 20, *P* = 0.035). Data are shown as box and whisker plots. Horizontal lines represent median values. The boxes encompass the first and 3rd quartile of the included data. The bars give the 95% confidence interval of the values. *P < 0.05.

 We examined the clinical effects of KRG on symptomatic IDH. The number of sessions disturbed by symptomatic IDH reduced significantly from 3.9 ± 3.1 times (per 12 times) to 2.7 ± 3.1 times in group A (*P* = 0.016) and from 3.9 ± 3.9 times to 2.7 ± 3.5 times in group B (*P* = 0.035) (Figure 2). The average pre- and postdialysis body weight, dry weight, and the amount of actual ultrafiltration were not significantly different between two study phases (Table 2). Thus, we could exclude the effect of altered ultrafiltration amount that might have affected the occurrence of IDH.

### 3.3. Effect of KRG on Blood Pressure during Hemodialysis

We looked at the effects of KRG in regulating the blood pressure changes during hemodialysis (Table 3). We found that the nadir SBP in group B was significantly elevated from 76.3 ± 16.2 mmHg in phase I to 79.1 ± 15.8 mmHg in phase II (*P* = 0.045). A similar trend of increased nadir SBP was noted in group A (97.4 ± 16.0 in phase I versus 100.1 ± 18.6 in phase II; *P* = 0.184). However, the administration of KRG did not significantly change prehemodialysis or posthemodialysis blood pressure.

Furthermore, we calculated the differences of blood pressure measured at different time points (prehemodialysis to nadir and to posthemodialysis) (Table 4). Notably, we found that patients in group B experienced less blood pressure changes during phase II (−24.4 ± 10.1 mmHg of Nadir-Pre SBP in phase I to −20.4 ± 11.0 mmHg in phase II, *P* = 0.045; −13.2 ± 7.1 mmHg of nadir-pre-DBP in phase I to −10.4 ± 6.6 mmHg in phase II, *P* = 0.011; −16.9 ± 7.1 mmHg of nadir-pre-MAP in phase I to −13.8 ± 7.7 mmHg in phase II, *P* = 0.015; −11.0 ± 6.3 mmHg to −7.8 ± 6.3 mmHg in post-pre-DBP, *P* = 0.025). A similar trend was also noted in group A after KRG treatment (−50.7 ± 39.3 mmHg of nadir-pre-SBP in phase I to −42.0 ± 23.8 mmHg in phase II, *P* = 0.053; −40.3 ± 39.3 mmHg of nadir-pre-MAP in phase I to −31.8 ± 24.8 mmHg in phase II, *P* = 0.02). When all patients were included for analysis, the blood pressure drop reduced significantly in the KRG-treated period (−36.9 ± 30.7 mmHg of nadir-pre-SBP in phase I to −31.0 ± 21.3 mmHg in phase II, *P* = 0.006; −22.4 ± 14.5 mmHg of nadir-pre-MAP in phase I to −19.3 ± 12.7 mmHg in phase II, *P* = 0.004). These results indicate that KRG may help to keep hemodynamic stability in IDH patients.

### 3.4. Effect of KRG on PRA, ET-1, AngII, and NT

To investigate the action mechanism of KRG, we examined the plasma levels of PRA, ET-1, AngII, and NT (nitrite + nitrate). In the observation phase (phase I), PRA levels increased significantly after hemodialysis in group B (*P* = 0.004) but less evident in group A (*P* = 0.071). The level of ET-1 and AngII did not significantly increase despite an average ultrafiltration of 2.6 ± 1.0 kg with removal of fluid in both groups (*P* > 0.05).

**Figure 3:**
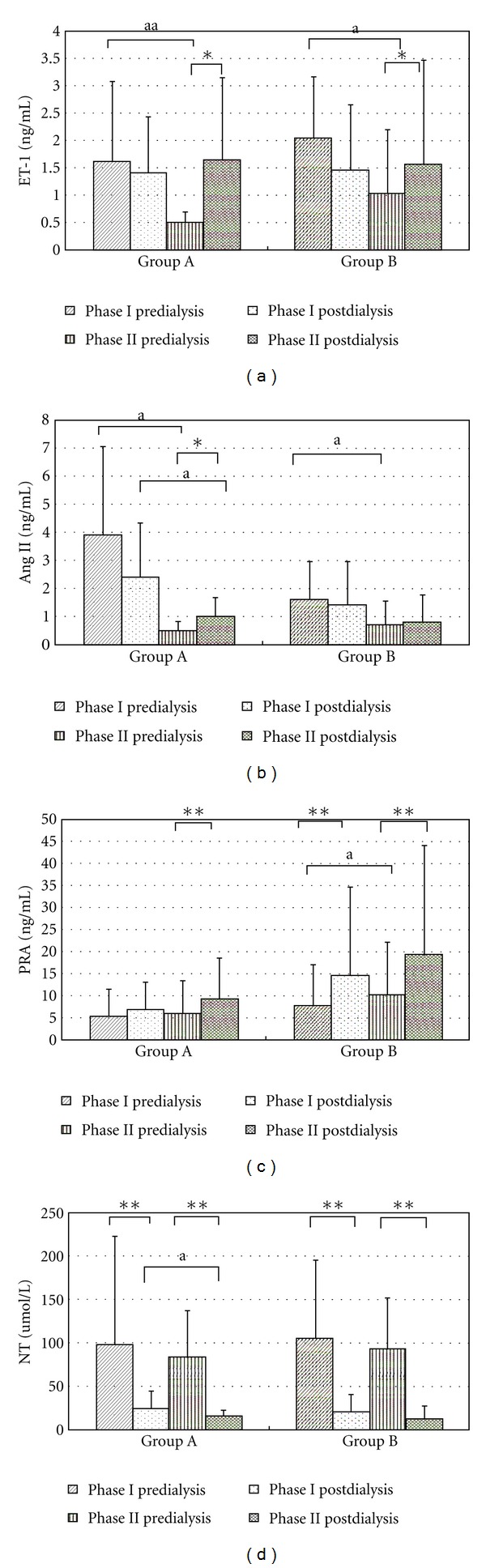
Changes of endotheline-1 (ET-1), plasma renin activity (PRA), angiotensin II (AngII), and NT (Nitrate+nitrite) plasma levels after KRG treatment in patients with intradialytic hypotension. Phase I is the control phase, and phase II is the KRG treatment phase. The ET-1 levels increased significantly after hemodialysis in phase II in both group A (hypertensive at baseline, *n* = 18, *P* = 0.035) and group B (normotensive or hypotensive at baseline, *n* = 20, *P* = 0.011). The posthemodialysis PRA levels increased significantly in phase II in both group A (*P* = 0.005) and group B (*P* < 0.001). In group A, the AngII levels significantly elevated after KRG treatment (*P* = 0.033). The levels of NT decreased significantly after dialysis in all groups in both phases (*P* < 0.0001). In group A, the posthemodialysis NT level was significantly lower in phase II than those in phase I (*P* = 0.028). *P < 0.05; **P < 0.01; ^a^ *P* < 0.05; ^aa^ *P* < 0.01.

After four weeks of KRG treatment (phase II), the posthemodialysis PRA levels increased significantly in both groups (*P* = 0.005 in group A, *P* < 0.001 in group B) (Figure 3). Similarly, the posthemodialysis ET-1 levels increased significantly by one- to threefold compared to the prehemodialysis levels (*P* = 0.035 in group A, *P* = 0.011 in group B), which is in contrast to the trend in phase I (Figure 3). The levels of AngII also significantly increased after dialysis in group A (*P* = 0.033), but not in group B (*P* = 1.000). A previous study has shown that NT can be removed by hemodialysis [4]. As expected, the levels of NT decreased significantly after dialysis in all groups. Nevertheless, the posthemodialysis NT levels which would cause vasodilation were significantly lower in the KRG treatment phase than those in the observatory phase (*P* < 0.05 in group A) (Figure 3). These results suggest that KRG treatment may improve the compensatory response mediated by various vasoconstrictors to acute volume change during hemodialysis.

Unexpectedly, we observed a two- to threefold declination of prehemodialysis ET-1 levels in both groups with KRG treatment (1.627 ± 1.460 to 0.497 ± 0.202 ng/mL in group A, *P* = 0.014; 2.042 ± 1.127 to 1.023 ± 1.190 ng/mL in group B, *P* = 0.047) as shown in Figure 3. The same holds true for the comparison of prehemodialysis AngII levels between two phases (3.902 ± 3.162 to 0.477 ± 0.353 ng/mL in group A, *P* < 0.001; 1.616 ± 1.387 to 0.694 ± 0.859 ng/mL in group B, *P* < 0.001). In contrast, the predialysis PRA levels remained unchanged in the group A but elevated in group B (*P* = 0.012) with KRG treatment. There were no significant changes in the prehemodialysis NT levels during the two study phases.

### 3.5. Side Effects

No significant adverse effects were observed during the study. Side effects reported by patients were palpitation (*n* = 1 in group A; *n* = 1 in group B) and thirsty (*n* = 1 in group B). Serum potassium levels increased slightly from 4.3 ± 0.5 to 4.6 ± 0.7 mEq/L (*P* = 0.016) in group B with KRG administration, but the difference were not significant when data from both groups was pooled (4.4 ± 0.8 versus 4.6 ± 0.7 mEq/L, *P* = 0.189) (Table 5). Serum phosphate levels were slightly elevated from 4.9 ± 1.4 to 5.3 ± 1.6 mg/dL (*P* = 0.04) with KRG treatment, but the levels were still within the normal range. No other significant changes in hematologic or biochemical parameters were observed (Table 5).

## 4. Discussion

The current treatments for IDH include stopping ultrafiltration, increasing dialysate sodium and glucose concentrations, and administration of hypertonic solutions and vasoconstrictor agents [1, 6, 12–14]. In the current study, we found that taking 3.5 g KRG slices at the start of hemodialysis can elevate the nadir blood pressure and reduce the frequency of symptomatic IDH by increasing the nadir blood pressure. KRG had no significant effects on the baseline blood pressure, suggesting that its beneficial effect is through restoring the vasoconstrictive response to acute plasma changes during hemodialysis. Furthermore, KRG has been used in different pathologic conditions [15], and its safety profile has been well studied in patients with normal renal function [16]. Our results confirmed the safety of using oral form KRG during hemodialysis. Although KRG has been reported to increase blood pressure in non-dialysis patients [17–19, 26, 27], we did not observe exacerbation of preexisting hypertension in our patients (group A). Our results suggest that KRG may be an alternative and adjuvant treatment for IDH.

The effects of *Panax ginseng* on regulating blood pressure were controversial due to its complexity of major components and different actions in various pathological conditions [18, 19, 22, 28, 29]. It has been reported that dammarenetriol glycosides in *Panax ginseng* have strong CNS excitatory actions that may cause hypertension, while dammarenediol glycosides in *Panax ginseng* have sedative and antihypertensive effects [26]. Three decades ago, Siegel proposed that low-dose *Panax ginseng* could cause hypertension [18]. However, high-dose *Panax ginseng* was shown to increase NO production in recent clinical trials and laboratory experiments [29], which may reduce blood pressure [28, 30]. Furthermore, *Panax ginseng* induced different responses in different blood vessels taken from rabbits, dogs and humans qualitatively and quantitatively [31]. Our data suggest that low-dose KRG (3.5 gm per dialysis session) could maintain the stability of blood pressure rather than exacerbating hypotension during hemodialysis.

The mechanism of IDH has been partly attributed to endothelial dysfunction in response to hemodynamic instability, with increased NO and decreased ET-1 during hemodialysis [4, 32–34]. ET-1 is the most potent vasoconstrictor that is locally produced from vascular endothelial cell [35, 36]. ET-1 levels decrease in IDH prone patients and increase in patients who have hypertension during hemodialysis, implying its importance in regulating hemodynamic stability [4]. Consistently, we did not found a significant increase of ET-1 levels during hemodialysis in these hypotension-prone patients during the observation period, suggesting a lack of adequate vasoconstrictive response. Indeed, we detected a significant increase in ET-1 levels during hemodialysis after KRG treatment. Similarly, we found that KRG treatment led to more activation of PRA and AngII during hemodialysis, indicating a gradually restoration of neurohormonal and cardiovascular responses to acute plasma volume change. Although NT concentrations is an indirect measurement of NO and can be removed by dialysis [4], we observed a significant decrease in the posthemodialysis NT levels after KRG treatment (group A). The result suggested that the beneficial effects of KRG treatment on IDH may be partially due to decreased NO production.

Previous studies have shown that hypotension-prone patients have higher baseline AngII levels compared to hypotension resistant dialysis patients [37]. This indicates that renin-angiotensin-aldosterone system may be abnormally activated in patients with recurrent IDH but still incapable to have adequate cardiovascular capacitance to the dialytic ultrafiltration. Elevation of AngII may lead to endothelial dysfunction and increase the risk of adverse cardiovascular events [38]. In the current study, we found that KRG treatment resulted in significantly reduced baseline AngII and ET-1. Our data suggest that KRG may have additional beneficial effects on endothelial dysfunction in patients with IDH by lowering the baseline of ET-1 and AngII levels [36, 39].

Despite the promising results obtained in this trial, several limitations should be acknowledged. First, the study had a relative small sample size, and further studies are needed to confirm the results. Second, the study is a phase I pilot study, and we could only observe the differences within the same patient although this also help to eliminate the variation of blood pressure changes among different patients. Third, the long-term effects of KRG in hemodialysis patients were not studied, and further investigations are warranted.

## 5. Conclusions

Chewing low-dose KRG renders patients better resistance to acute BP reduction during hemodialysis. KRG treatment improves the compensatory response to acute volume change during hemodialysis via activation of vasoconstrictors (ET-1 and AngII). Our results suggest that KRG could be an adjuvant treatment for IDH.

## Figures and Tables

**Table 1:** Baseline characteristics of study patients.

	Total (*n* = 38)	Group A (HTN) (*n* = 18)	Group B (Non-HTN) (*n* = 20)	*P* value
Age (year)	52.6 ± 12.4	56.2 ± 12.7	49.3 ± 11.5	0.083
Sex (% Female)	25 (65.8%)	11 (61.1%)	14 (70%)	0.734
Dry BW (kg)	60.7 ± 16.6	62.5 ± 15.0	59.0 ± 18.1	0.515
BMI	23.6 ± 5.2	24.1 ± 4.2	23.1 ± 6	0.579
Time on dialysis (months)	93.0 ± 61.9	67.9 ± 48.7	115.5 ± 64.9	0.016*

*Comorbidities*				
DM	15 (39.5%)	13 (72.2%)	2 (10%)	<0.001**
HTN history	26 (68.4%)	18 (100%)	8 (40%)	<0.001**
Previous MI	1 (2.6%)	1 (5.6%)	0 (%)	0.474
Angina history	5 (13.2%)	3 (16.7%)	2 (10%)	0.653

Values are expressed as means ± SD or number (%); BMI, body mass index; BW, body weight; DM, diabetes mellitus; HTN, hypertension; MI, myocardial infarction; *P < 0.05; **P < 0.01.

**Table 2:** Comparison of body weights and ultrafiltration rates with and without Korean red ginseng treatment.

	Group A (HTN)		*P* value	Group B (non-HTN)		*P* value	All		*P* value
	Phase I (−KRG)	Phase II (+KRG)		Phase I (−KRG)	Phase II (+KRG)		Phase I (−KRG)	Phase II (+KRG)	
BW predialysis (Kg)	65.3 ± 15.5	65.4 ± 15.4	0.862	61.6 ± 18.8	61.7 ± 18.8	0.218	63.4 ± 17.2	63.4 ± 17.1	0.415
BW postdialysis (Kg)	62.7 ± 15.0	62.8 ± 15.0	0.296	59.0 ± 18.1	58.9 ± 18.1	0.888	60.7 ± 16.6	60.7 ± 16.6	0.994
Dry BW (Kg)	62.5 ± 15.0	62.6 ± 15.0	0.054	59.0 ± 18.1	59.0 ± 18.2	0.386	60.7 ± 16.6	60.7 ± 16.6	0.282
Target UF (L)	2.6 ± 1.0	2.6 ± 0.9	0.616	2.7 ± 1.0	2.8 ± 1.1	0.322	2.6 ± 1.0	2.7 ± 1.0	0.33
Actual UF (L)	2.8 ± 1.1	2.7 ± 1.0	0.850	2.7 ± 1.1	3.0 ± 1.6	0.198	2.7 ± 1.1	2.9 ± 1.4	0.338
% target UF	100.0 ± 21.5	106.4 ± 39.1	0.231	103.8 ± 10.4	103.0 ± 10.6	0.794	102.0 ± 16.5	104.6 ± 27.6	0.319

Values are expressed as means ± SD; BW, body weight; KRG, Korean red ginseng; UF, ultrafiltration; % target UF, percentage of actual UF/target UF. Phase I is the controlled phase without KRG treatment, expressed as −KRG; phase II is the KRG treatment phase, expressed as +KRG.

**Table 3:** Comparison of blood pressure measurements during hemodialysis with and without Korean red ginseng treatment.

	Group A (HTN)		*P* value	Group B (non-HTN)		*P* value
	Phase I (−KRG)	Phase II (+KRG)		Phase I (−KRG)	Phase II (+KRG)	
Pre- SBP (mmHg)	148.1 ± 39.3	142.9 ± 30.8	0.231	100.8 ± 16.9	99.5 ± 14.5	0.332
Pre-DBP (mmHg)	71.1 ± 10.4	71.3 ± 13.0	0.931	58.2 ± 9.7	56.8 ± 8.1	0.279
Pre-MAP (mmHg)	96.8 ± 18.7	95.2 ± 18.5	0.327	72.4 ± 11.9	71.0 ± 9.9	0.218
Nadir SBP (mmHg)	97.4 ± 16.0	100.1 ± 18.6	0.184	76.3 ± 16.2	79.1 ± 15.8	0.045^a^
Nadir DBP (mmHg)	53.9 ± 8.4	54.8 ± 9.4	0.338	45.0 ± 9.8	46.4 ± 10.3	0.341
Nadir MAP (mmHg)	68.4 ± 10.4	69.9 ± 11.9	0.231	55.5 ± 11.8	57.2 ± 12.02	0.296
Post-SBP (mmHg)	107.8 ± 17.2	111.1 ± 17.2	0.053	86.7 ± 30.9	84.8 ± 16.4	0.305
Post-DBP (mmHg)	58.4 ± 8.7	59.0 ± 8.8	0.396	47.2 ± 10.1	49.0 ± 10.9	0.135
Post-MAP (mmHg)	74.9 ± 10.7	76.4 ± 11.1	0.248	60.3 ± 16.3	61 ± 12.3	0.284

Values are expressed as means ± SD of the data; KRG: Korean red ginseng; pre-: prehemodialysis; post-: posthemodialysis; SBP: systolic blood pressure; DBP: diastolic blood pressure; MAP: mean arterial pressure; ^a^ *P* < 0.05.

**Table 4:** Changes of blood pressure during hemodialysis with and without Korean red ginseng treatment.

	Group A (HTN)		*P* value	Group B (non-HTN)		*P* value	All		*P* value
	Phase I (−KRG)	Phase II (+KRG)		Phase I (−KRG)	Phase II (+KRG)		Phase I (−KRG)	Phase II (+KRG)	
ΔNadir-pre-SBP (mmHg)	−50.7 ± 39.3	−42.9 ± 23.8	0.053	−24.4 ± 10.1	−20.4 ± 11.0	0.045*	−36.9 ± 30.7	−31.0 ± 21.3	0.006**
ΔNadir-pre-DBP (mmHg)	−17.3 ± 8.8	−16.6 ± 10.0	0.446	−13.2 ± 6.2	−10.4 ± 6.6	0.011*	−15.1 ± 7.7	−13.3 ± 8.8	0.020*
ΔNadir-pre-MAP (mmHg)	−28.4 ± 18.0	−25.4 ± 14.4	0.078	−16.9 ± 7.1	−13.8 ± 7.7	0.015*	−22.4 ± 14.5	−19.3 ± 12.7	0.004**
ΔPost-preSBP (mmHg)	−40.3 ± 39.3	−31.8 ± 24.8	0.020*	−14.1 ± 23.4	−14.7 ± 9.9	0.232	−26.5 ± 34.2	−22.8 ± 20.2	0.011*
ΔPost-preDBP (mmHg)	−12.7 ± 10.1	−12.3 ± 10.3	0.408	−11.0 ± 6.3	−7.8 ± 6.3	0.025*	−11.8 ± 8.2	−10.0 ± 8.6	0.027*
ΔPost-preMAP (mmHg)	−21.9 ± 18.6	−18.8 ± 14.9	0.048*	−12.0 ± 10.3	−10.1 ± 7.1	0.145	−16.7 ± 15.4	−14.2 ± 12.1	0.014*

Values are expressed as means ± SD of the data.

Pre-: prehemodialysis; post-: posthemodialysis; SBP: systolic blood pressure; DBP: diastolic blood pressure; MAP: mean arterial pressure;

*P < 0.05; **P < 0.01; Δ: changes.

**Table 5:** Comparison of laboratory parameters with and without Korean red ginseng treatment.

	Group A (HTN)		*P* value	Group B (non-HTN)		*P* value	All		*P* value
	Phase I (−KRG)	Phase II (+KRG)		Phase I (−KRG)	Phase II (+KRG)		Phase I (−KRG)	Phase II (+KRG)	
Hct (%)	32.5 ± 3.6	33.2 ± 3.3	0.170	33.3 ± 3.2	32.9 ± 3.6	0.537	32.9 ± 3.4	33.0 ± 3.4	0.795
Hb (g/dL)	10.7 ± 1.1	10.8 ± 1.0	0.276	10.6 ± 1.1	10.5 ± 1.2	0.359	10.7 ± 1.1	10.6 ± 1.1	0.849
Ca (mg/dL)	9.7 ± 1.3	9.6 ± 0.8	0.938	10.1 ± 1.1	10.1 ± 1.2	0.618	9.9 ± 1.2	9.8 ± 1.0	0.543
P (mg/dL)	5.0 ± 1.7	5.6 ± 1.9	0.065	4.8 ± 1.0	5.1 ± 1.3	0.360	4.9 ± 1.4	5.3 ± 1.6	0.04*
K (mEq/L)	4.5 ± 1.0	4.5 ± 0.7	0.796	4.3 ± 0.5	4.6 ± 0.7	0.016*	4.4 ± 0.8	4.6 ± 0.7	0.189
BUN (mg/dL)	63.4 ± 22.1	64.3 ± 19.2	0.943	58.0 ± 11.0	62.2 ± 14.9	0.083	60.5 ± 17.2	63.2 ± 16.9	0.186
Cr (mg/dL)	10.1 ± 2.9	10.0 ± 2.7	0.523	11.3 ± 1.9	11.3 ± 2.2	0.985	10.7 ± 2.4	10.7 ± 2.5	0.768
ALT (U/L)	16.2 ± 10.0	14.7 ± 7.7	0.459	15.0 ± 6.2	13.8 ± 3.9	0.599	15.6 ± 8.1	14.2 ± 5.9	0.183

Values are expressed as means ± SD of the data.

Hct: hematocrit; Hb: hemoglobin; Ca: calcium; P: phosphate; K: potassium.

BUN: blood urea nitrogen; Cr: creatinine; ALT: alanine aminotransferase.

*P < 0.05; **P < 0.01.
